# A non-fungible token (NFT)-Based framework for antenatal care in resource-constrained settings

**DOI:** 10.1177/20552076261478842

**Published:** 2026-08-17

**Authors:** Khulekani Sibanda, Patrick Ndayizigamiye, Hossana Twinomurinzi

**Affiliations:** 1 Department of Applied Information Systems, 61799 University of Johannesburg, Johannesburg, South Africa; 2 Centre for Applied Data Science, 61799 University of Johannesburg, Johannesburg, South Africa; 3 Centre for Augmented Intelligence and Data Science, 56866 University of South Africa, Johannesburg, South Africa

**Keywords:** blockchain, non-fungible tokens (NFTs), maternal health records, patient agency, resource-constrained settings, Actor-Network Theory (ANT)

## Abstract

**Background:**

In many low-resource settings, maternal health records are paper-based, fragmented, and predominantly controlled by institutions. These conditions limit patient agency, disrupt continuity of care, and constrain the efficiency of service delivery.

**Objective:**

This study examines the potential of blockchain technology, specifically non-fungible tokens (NFTs), to support a patient-centred approach to antenatal and postnatal record management.

**Methods:**

Drawing on Actor-Network Theory (ANT), we conducted a qualitative case study involving eight semi-structured stakeholder interviews at primary healthcare facilities in Zimbabwe. The themes that emerged from the qualitative enquiry were triangulated with observational data and findings from a previously conducted and published systematic literature review by the authors. This multi-method design enabled the exploration of both technical and socio-organisational factors shaping maternal record practices.

**Results:**

The study identified persistent challenges, including record duplication, limited interoperability, weak referral mechanisms, and inadequate processes for patient consent and data control. To address these gaps, an NFT-based conceptual architectural framework is proposed that tokenises record ownership and consent for data sharing. This framework has the potential to enable patients to exercise greater agency by controlling access to their data while allowing providers to be granted secure, time-bound permissions tailored to their clinical needs. NFTs also have the potential to reconfigure socio-technical networks by aligning stakeholder interests, strengthening patient agency, and supporting sustainable mobilisation across the health system.

**Conclusions:**

The proposed architectural framework is intended to position women as active participants in their maternal care pathways and to strengthen continuity across encounters. By embedding ownership and consent into digital records, blockchain-enabled management offers a potential pathway to more secure, equitable, and patient-centred maternal health systems in low-resource contexts. Empirical validation through pilot implementation and stakeholder co-design is essential before deployment.

## Introduction

Electronic Health Records (EHRs) are critical for protecting patient privacy and ensuring data availability.^1,2^ However, in low-and middle-income countries(LMICs), fragmented, paper-based records severely constrain maternal healthcare, and a lack of integrated electronic systems disrupts care continuity and compromises patient safety.^3,4^ Furthermore, centralised data ownership models, in which hospitals control access, limit patients’ agency in managing their health information.^5^ While blockchain implementations in healthcare face well-documented challenges, including infrastructure requirements, governance complexity, and constraints on consent revocation, this study explores how these technologies might be adapted for LMIC contexts.^6–8^ To address these challenges, this study aimed to develop an architectural framework using blockchain technology and non-fungible tokens (NFTs) to enhance data security, streamline sharing, and empower patients to control their maternal health records in resource-constrained settings.

To translate the technological affordances of NFTs into contextualised healthcare solutions, this study employs Actor-Network Theory (ANT) as a guiding framework. By considering the agency of both human actors (patients and healthcare providers) and non-human actors (technology and infrastructure), ANT offers a valuable lens for examining stakeholder dynamics and the socio-technical integration of blockchain and NFTs into EHRs.^9^

Specifically, this study sought to answer the following research questions (RQ):

• RQ1: What are the current medical record management deficiencies in antenatal and postnatal care in low-resource settings?
• RQ2: How do key antenatal and postnatal care system actants interact within the current record management practices?
• RQ3: How can an NFT-based architectural framework be designed for antenatal and postnatal care in resource-constrained settings?

This research is framed as a qualitative, exploratory case study designed to inform a conceptual architectural framework, rather than empirically testing a deployed system. This study makes three contributions. First, it expands research on maternal healthcare records by proposing a novel conceptualisation of maternal records as patient-owned NFTs with portable, time-bound consent, operationalising patient agency alongside interoperability standards such as Fast Healthcare Interoperability Resources (FHIR).^10^ Second, from a practical standpoint, it presents a conceptual architectural framework supporting decentralised record ownership and controlled access. Third, it extends ANT to decentralised systems. Notably, we present this as a theoretical proposition: NFTs have the potential to reconfigure socio-technical networks by shifting pregnant women from passive subjects to active data owners, thereby granting them verifiable authority over their records.^11^

## Literature review

### Challenges in maternal healthcare record management in LMICs

Maternal healthcare record management in LMICs faces interconnected challenges that undermine the continuity of care, data quality, and patient participation. Continuity of care is essential for maternal health, particularly for identifying high-risk pregnancies across facilities and over time. However, fragmented health information systems, episodic care delivery, and poor referral pathways exist.^3,12,13^ Paper-based records remain dominant; patients must recount medical histories at each new facility, while registers are poorly maintained, consume substantial consultation time, and make retrieval difficult, and patient-held cards are frequently misplaced or damaged.^5,14–16^ Although some institutions have adopted EHRs, they typically operate in isolation, with limited inter-facility sharing, and referrals still rely on handwritten notes, impeding the transmission of critical data.^4,5^

Beyond infrastructural gaps, health data governance and security frameworks remain weak, with underdeveloped privacy safeguards, limited standardisation of consent protocols, and poor patient access to digital records, limiting patient agency.^8,17,18^ Established digital health platforms, such as OpenMRS, have successfully digitised clinical records in various LMIC settings, including Zimbabwe,^19^ yet they remain largely provider-centric. This often limits patients to the role of passive data subjects. Even interoperability standards, such as FHIR, which improve data exchange, lack native support for data sovereignty.^20^

### Patient agency and access to records

Patient agency, defined as the ability to actively participate in healthcare decisions, understand options, and advocate for oneself,^21^ is fostered through patient-centred approaches that prioritise patients’ needs and involve them in decision-making.^22,23^ Digital tools, such as patient portals, support this by providing access to health data and facilitating cooperation between patients and practitioners.^24,25^ Research indicates that enhanced autonomy improves outcomes, satisfaction, and cost efficiency; specifically, maternal self-efficacy is associated with better functional status in antenatal and postnatal care.^23^ Crucially, exercising this agency requires reliable access to health information and collaboration within the healthcare system.^5,26,27^

### NFTS in healthcare

Blockchain-based EHR solutions empower patients to manage access to their medical records while ensuring data integrity.^28–30^ Within this domain, NFTs have emerged as a key mechanism for patient-centric data management.^29^ By representing ownership rights as unique tokens, NFTs support granular consent and accountability through smart contracts, allowing patients to grant specific access to authorised parties while preserving an immutable audit trail.^8,31^

While earlier blockchain initiatives utilised permissioned ledgers to bolster data integrity and provide audit trails,^7^ they often conceptualised health records as static entries, lacking the flexibility to manage medical information as dynamic, patient-governed assets. Our proposed architectural framework addresses this limitation by leveraging NFTs to “assetise” the maternal care pathway. By minting specific clinical milestones, such as a 24-week ultrasound or an immunisation event, as unique tokens, the medical record transforms into a portable, non-interchangeable entity. This distinction facilitates granular, programmable consent linked to the patient across fragmented health networks^8^ and establishes a foundation for an incentive marketplace that rewards adherence milestones in ways that conventional databases or fungible ledgers cannot replicate.^32^

However, the current literature indicates that much of the research on NFTs in healthcare, including frameworks proposing incentive marketplaces, remains conceptual, with a notable lack of empirically validated, deployed studies in clinical settings.^7^ The literature frequently underrepresents the risks and failures of real-world blockchain implementation. Especially in resource-constrained settings, NFT architectures face significant practical hurdles, including infrastructure and transaction costs, technical demands on clinicians and patients, and governance complexities for decentralised networks. Critical concerns persist about the potential to widen existing digital divides where connectivity is unreliable and digital literacy is limited.^6,7^

The transition to blockchain also introduces ethical, legal, and governance critiques that are often overlooked in conceptual models. For instance, the literature highlights that the immutability of blockchain data (data permanence) inherently conflicts with patients’ rights to data correction, effective consent revocation, or the legal ‘right to be forgotten'.^7,8,33^ Finally, there is a risk of exacerbating power asymmetries; if governance mechanisms are weak, empowering economically vulnerable pregnant women to monetise their health data through NFT marketplaces could enable exploitation by institutional or commercial consumers.^23,32,34^

Beyond the permissioned blockchain and self-sovereign identity (SSI) approaches, other decentralised routes to patient-controlled data include decentralised identifiers,^35^ verifiable credentials,^36^ and dedicated consent-management platforms.^37^ These approaches are strong at authentication and at expressing who may access what, but they characteristically represent the health record as a credential or a permission rather than as an ownable, transferable asset, and they offer no native mechanism for linking consent to programmable incentives. A gap therefore remains: no established approach simultaneously provides encounter-level ownership with provenance and integrated, consent-linked incentives, the combination most relevant when the aim is to reposition patients from data subjects to data owners. This gap motivates the present study’s exploration of NFTs, whose comparative characteristics are examined in the Discussion (Table 7).

Notwithstanding these risks, by foregrounding patient agency, granting pregnant women control over their medical data, and enabling selective, temporary access to authorised providers, the proposed framework fills a critical gap in existing digital health interventions, which often overlook patient empowerment in these settings.^23^

### Actor-Network Theory in health information systems

Actor-Network Theory (ANT), developed by Callon, Latour, and Law, provides a relational framework for examining how heterogeneous networks of human and non-human actors are assembled, stabilised, and transformed.^38^ Central to ANT is the principle of generalised symmetry, which treats human actors, such as patients and clinicians, and non-human actors, such as technologies, documents, and institutional protocols, as equally capable of shaping network dynamics.^39^ ANT conceptualises network formation through four moments of translation: *problematisation*, *interessement*, *enrolment*, and *mobilisation.*^38,40^ These concepts have been applied in health information systems research to examine how technical implementations negotiate social and organisational forces, including EHR adoption in primary healthcare^9^ and blockchain deployment in government services in resource-constrained settings.^40^ In the context of this study, ANT provides a theoretical lens for examining how a heterogeneous network of organisational, human, and technological actants interacts within the current maternal healthcare system, and how the introduction of NFT infrastructure, as a new non-human actant, has the potential to reconfigure those relationships. Table 1 outlines the potential contributions of ANT to the proposed NFT-based implementation in maternal healthcare.

**Table 1.** Potential contributions of ANT in implementing NFTs in maternal healthcare.

Key notion	Contribution of this notion	Implications for our study
Role of actors	It explains how non-human actors transform human actors and influence their associations.	Positions NFTs, blockchains, and other non-human actors as active agents transforming maternal healthcare practices rather than as passive entities.
Problematisation	It defines the initial framing of the problem and the indispensable role of the proposed solution.	Frames the current fragmentation of maternal records as a critical failure and establishes the concept of NFT-based verifiable ownership as the necessary passage point to resolve security and access issues.
Interessement	Strategies are used to lock allies into place and disrupt competing associations.	It involves negotiating a consensus around the shared goal of improving maternal healthcare through NFTs and aligning diverse actors, including healthcare providers, patients, policymakers, and technology developers, to explore the potential benefits of NFTs.
Enrolment	It explores how different actors become part of the network.	Describes the technical and social construction of the network: minting permissions to maternal health records, medical images, and other relevant data as tokens and deploying smart contracts to govern access, designed to lock human and nonhuman actors into defined roles effectively.
Mobilisation	This process occurs when certain actors successfully speak or act on behalf of others, ensuring that the network functions as intended.	The NFT-based system stores a patient’s health record and aggregates and mobilises diverse data, including medical history, antenatal visits, and referrals, thereby representing the patient’s clinical history.

## Methods

This study adhered to the Standards for Reporting Qualitative Research (SRQR).^41^ Ethical approval was obtained from the host university. Written and verbal informed consent was obtained from participating institutions and individual participants prior to the interviews, and all transcripts were de-identified. To ensure trustworthiness, transferability was supported by detailed contextual descriptions, dependability through an audit trail of decisions, notes, and analyses, and conformability through reflexive journaling and direct quotations.^42^

Consistent with this conceptual framing, the study integrates theoretical insights and empirical data on stakeholder dynamics to ensure that NFT-enabled innovations align with the operational realities of maternal healthcare in Zimbabwe. This multiphase methodology prioritises a sociotechnical understanding before proposing a final architecture and comprises two distinct phases.

### Systematic literature review

First, a systematic literature review was conducted to map global research on NFTs in healthcare, with findings published in.^29^ This review aimed to identify existing macro-level gaps and technical requirements for blockchain applications in medical record management. These theoretical insights provide the foundational design requirements for the proposed architectural framework. They were subsequently triangulated with micro-level operational challenges identified during the fieldwork phase to ensure that the final design addressed both technical gaps and clinical realities.

### Case study

Before presenting the case study results, it is important to demarcate the analytical boundaries of this study. As a qualitative, exploratory case study focused on mapping socio-technical workflows and stakeholder interactions, this study relied entirely on thematic analysis of interview data and literature. Statistical modelling or quantitative analyses fall outside the scope of this study and will be applied in future research to assess the potential acceptance and adoption of the resultant NFT artefact.

### Analytical framework

ANT was operationalised as the primary theoretical and analytical lens for both data collection and thematic analysis. Drawing on established practice in health informatics research,^38,40^ the four ANT translation moments, problematisation, interessement, enrolment, and mobilisation, served as the deductive analytical categories through which the thematic analysis was structured and interpreted. Participants’ accounts were coded by mapping reported experiences onto these moments: systemic failures and workflow frustrations were assigned to problematisation; stakeholder negotiations and collaborative arrangements were assigned to interessement; role assignments and workaround practices were assigned to enrolment; and information flow patterns and communication breakdowns were assigned to mobilisation.

**Figure 1.**
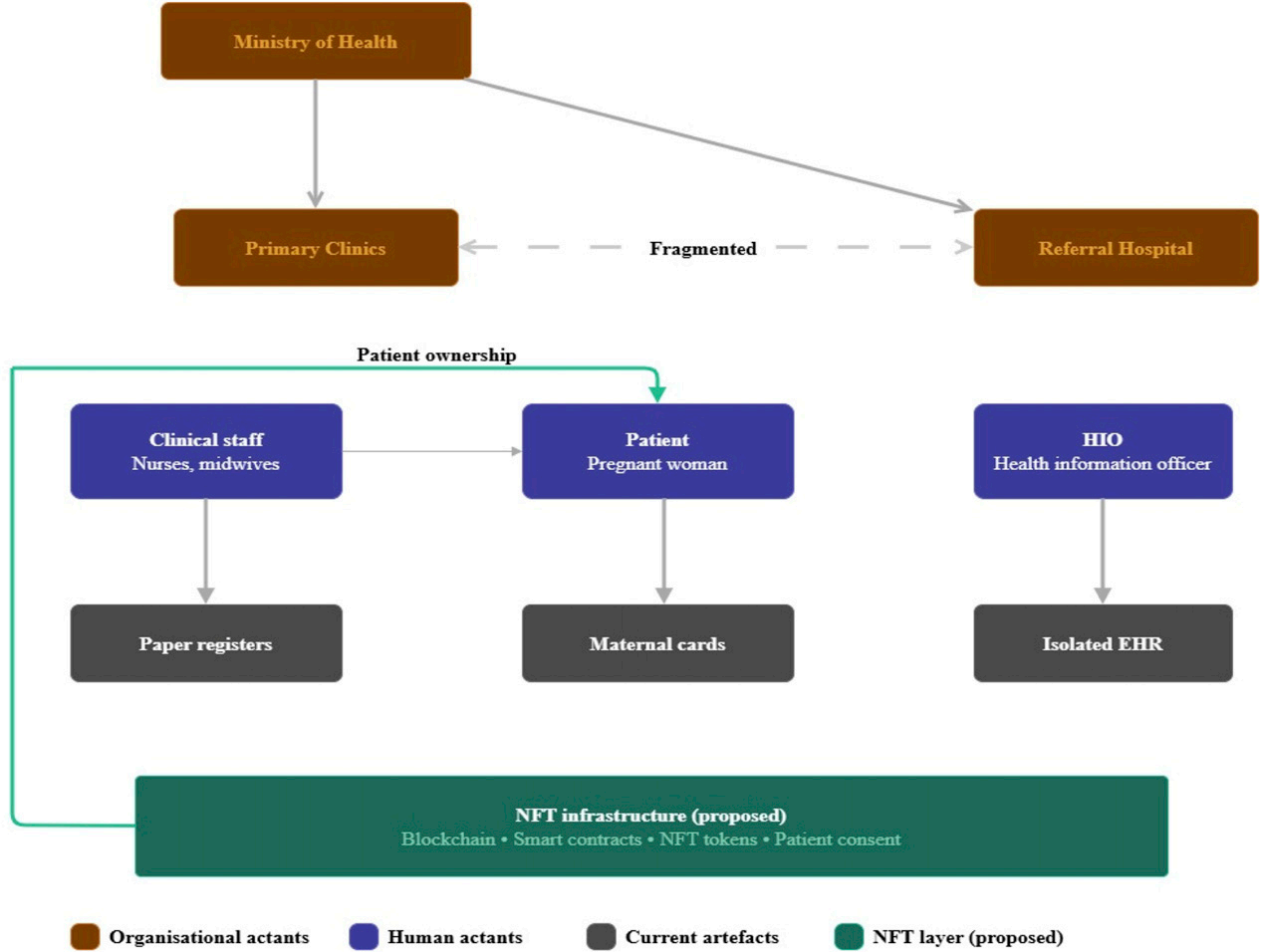
Socio-technical network of identified stakeholders and the proposed NFT-based actants in maternal healthcare.

Actants were identified across three categories relevant to the maternal healthcare context under study. Human actants included midwives, nurses, a sister-in-charge, a doctor, health information officers, and patients (pregnant women). Organisational actants encompassed primary clinics, the referral hospital and the Ministry of Health. Technological actants comprised paper-based maternal registers, maternal health cards, electronic health record systems, and the proposed NFT-based infrastructure. Figure 1 illustrates the actor network specific to this study, mapping the relationships between these actants within the current system and the proposed NFT-enabled reconfiguration. Notably, whereas patients were not directly interviewed, they serve as the central actant of the proposed framework, the entity whose repositioning from passive data subject to active data owner motivates the entire NFT architecture.

### Field work and stakeholder engagement

The second phase involved fieldwork at a public regional referral hospital and local council clinics in Zimbabwe. These sites were selected to capture the flow of care from the initial registration to emergency referral. Purposive sampling was used to recruit participants with expertise relevant to the study.^43^ Eight (8) participants were selected, each with practical experience with both paper-based and electronic record-keeping systems used in antenatal and postnatal care. The sample included sisters-in-charge (clinical administrators), midwives (specialist nurses involved in antenatal and postnatal services), and health information officers. Semi-structured interviews were conducted with all eight stakeholders in this study. To protect their confidentiality, the participants are identified using pseudonyms (P1-P8) throughout the findings. Table 2 summarises the participants’ profiles. While the sample size is focused, its adequacy is supported by the “Information Power” framework, which suggests that the depth of a sample is determined by the quality of the dialogue and the density of participants’ expertise.^44^ Given the professional background of some participants, such as P4, with 21 years of clinical experience, the data provided high analytical power for mapping the current actor network.

**Table 2.** Participants.

Participant’s ID	Role	Gender	Years of experience
P1	Sister in Charge	Female	3
P2	Midwife	Female	10
P3	Nurse	Female	11
P4	Midwife	Female	21
P5	Health Information Officer	Female	5
P6	Midwife	Male	5
P7	Midwife	Female	10
P8	Doctor	Male	2

Data collection involved semi-structured interviews conducted between August 2023 and April 2024. Due to constraints in the clinical environment, the sessions were focused and lasted 12-23 minutes. This format allowed for probing and clarifying complex clinical workflows while respecting participants’ duties. To strengthen credibility, the interview findings were triangulated with the systematic literature review findings and observations of record-keeping practices conducted concurrently with data collection. Following the interview sessions, the first author recorded brief reflective field notes documenting observed record-keeping behaviours, including how nurses and midwives recorded patient data during consultations and subsequently transcribed the information into registers or electronic systems. While unstructured and supplementary rather than primary data, these observations provided essential contextual grounding, confirming the labour-intensive nature of manual data duplication and the physical movement of maternal cards between facilities, and ensuring that challenges identified through interviews were consistent with the clinic’s operational realities. Theoretical saturation was confirmed during the thematic analysis in ATLAS.ti when the final two interviews yielded no new codes or conceptual categories.

The sampling scope of this study and its intentional focus on clinical and administrative personnel reflect a deliberate methodological decision aligned with its exploratory nature. Establishing a supply-side baseline, mapping institutional workflows, identifying register redundancies, and documenting referral pathways and connectivity constraints are essential prerequisites for any subsequent system design.^45^ This sequencing follows established co-design frameworks in health informatics, which prioritise provider-side workflow mapping in the early phases before patient engagement in subsequent co-design cycles.^45^ Consistent with this approach, while patient agency is the ultimate objective of the proposed architectural framework, direct patient engagement was deferred to future co-design cycles, where participants can respond to concrete system prototypes rather than abstract propositions, reflecting how health consumer involvement is reserved for later translational phases once workflow realities are well understood. Similarly, macro-level actors, policymakers, regulators, and IT system vendors are critical to governance and deployment but fall outside the micro-level clinical focus of this study. Engagement with these actor groups is planned for subsequent regulatory feasibility and pilot testing.

## Case study context: Maternal referral system and antenatal care in Zimbabwe

### Maternal referral system in Zimbabwe

The healthcare referral system in Zimbabwe is hindered by poor coordination among the centres. Notably, a study conducted at two major Harare referral hospitals found that while 81.6% of mothers had referral notes from lower-level facilities, feedback on referral outcomes was often absent.^12^ Security vulnerabilities compound these operational challenges. Many EHR systems rely on weak password authentication,^5^ creating perceived insecurity that stifles widespread adoption.^46^ The World Health Organisation (WHO) echoes this, citing privacy risks as a significant barrier to mobile health implementation in Zimbabwe.^47^ Although Zimbabwe has adopted the District Health Information System version 2 (DHIS2), an open-source, web-based platform that supports data customisation, analysis, visualisation, and integration,^48^ paper-based maternity cards remain the primary means of tracking antenatal and postnatal care.

### Current antenatal CARE(ANC) process

**Figure 2.**
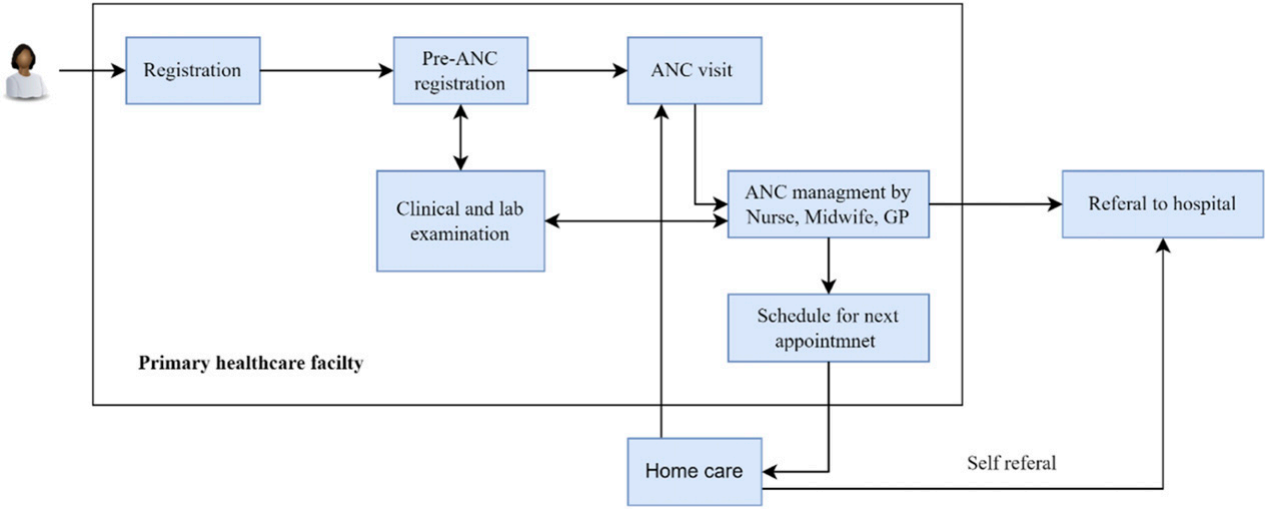
Routine ANC process in a primary healthcare facility (Adapted from^50,51^).

Zimbabwe’s healthcare system adheres to the WHO guidelines for ANC, which recommend that the first ANC visit occur within the first 12 weeks of pregnancy, followed by eight subsequent visits throughout the pregnancy.^49,50^ In this system, information typically flows hierarchically from lower- to higher-level healthcare facilities.^13^ Consequently, this section details the specific processes involved in delivering ANC services at the facility level, focusing on the associated information flow, as shown in Figure 2.

### Registration

Patients register at designated service delivery points. A healthcare professional identifies the patient by searching existing records or by creating a new profile, and demographic information is validated. Following payment, the patient’s encounter is, in some instances, documented in the patient’s handbook or card during a consultation with a clinician.

### ANC management and visits

During each visit, clinicians collect medical history, conduct physical examinations, and order routine or specialised laboratory tests to evaluate maternal and fetal health. Abnormal findings are identified, treatment plans (including potential referrals) are developed, and preventive measures are discussed. Crucially, the details of every encounter are recorded in the patient’s maternal booklet and subsequent appointments are scheduled.

### Home care

Between visits, pregnant women monitor their health at home. They may seek immediate care at a secondary facility if symptoms arise, although some women delay or skip subsequent visits until they are close to the delivery date.

## Mapping systematic literature findings to the conceptual design

To bridge the gap between theoretical capabilities and practical implementation, insights derived from the systematic literature review were mapped to the structural requirements of the proposed conceptual framework. The review revealed a critical lack of patient ownership models in resource-constrained settings.^29^ These insights provided the foundation for the architectural framework’s design requirements, which are systematically mapped to its components in Table 3.

**Table 3.** Mapping of systematic review findings to the architectural framework design.

Systematic review insights from^29^	Implication/Requirement	Framework element in conceptual design
It focuses on ownership but pays little attention to patient agency in resource-constrained settings.^52,53^	Patient autonomy and control over health data must be embedded.	Patient Smart Contract and NFT-based consent tokens enabling patients to grant/revoke access.
Limited real-world contextualisation in fragmented systems.^28^	There is a need for a context-specific design that accounts for diverse actors and workflows.	Clinician, Lab Assistant, and Admin Smart Contracts are integrated to reflect the fundamental roles in maternal healthcare.
The persistent challenges of data fragmentation, poor interoperability, and inefficiencies.^5,54,55^	There is a need for secure, scalable, and interoperable record management.	Blockchain for immutability and auditability, combined with off-chain storage for scalability and integration.
Weak referral systems and a lack of continuity in maternal care are also present.^56^	Longitudinal patient pathways and inter-facility communication must be supported.	Referral functionality and workflow support in the framework service layer.
Limited incentives for patient engagement in digital health systems.^32^	Patient retention and participation require improvement.	Incentive calculation module and potential integration with NFT marketplace for rewards.

## Empirical findings

Qualitative analysis employed ANT translation moments,^39^ to identify system deficiencies and stakeholder requirements. By analysing the data through ANT’s moments of translation, *problematisation*, *interessement*, *enrolmen*t, and *mobilisation*, we gained critical insights into the complex dynamics and challenges, the interactions among various actors, and the role of NFTs.^38^ Thematic analysis was conducted using ATLAS.ti software, following the six methodical steps outlined by Braun and Clarke^57^: (1) familiarising with the data, (2) generating initial codes, (3) searching for themes, (4) reviewing themes, (5) defining and naming themes, and (6) producing the report. We adopted a hybrid coding strategy to align the analysis with our theoretical framework. While open inductive coding was used to capture emerging patterns, ANT translation moments served as the primary deductive categories. For example, P3’s account of needing to consult multiple registers to retrieve information and the resulting duplication of effort were coded inductively as duplicate effort (C2) and record inaccessibility (C3) and assigned to the ANT moment of ‘problematisation’. This approach ensured that socio-technical interactions were integral to the data structure before interpretation.

## Thematic analysis findings

### Problematisation

Problematisation involves the process by which a focal actor defines a problem and assigns roles to other actors in the network. According to the interviewees, the healthcare facility primarily relied on a paper-based system supplemented by electronic registers, resulting in redundant data entry and inefficient management. A registered nurse remarked, *“We enter data in the paper-based registers and then in the electronic system.”(P1)* They further state their challenges with such a system: “*It’s time-consuming to copy data from the registers and write it into the system.” (P2)*

*P6 also* stated the challenges of duplicating data, “*Yes, the duplication is there. There is some kind of duplication. It was hoped that some registers would be phased out and replaced by the electronic recording system after some time. Presently, that has not been done*”

Another respondent highlighted the challenges encountered during system downtime, noting that manually recording large volumes of information increases the risk of data-entry errors:

*“There may be delays in data entry, especially when we are busy. There will be a lot of information, and I have to look for information from different registers. For instance, I have to go through different registers for the ART program and antenatal and postnatal care to access information. Additionally, electricity and network challenges can sometimes disrupt the system, making it difficult to access and enter patient information which leads to too much work transferring information from paper records into the system.” (P3)*

Although some data are recorded electronically, access is restricted to clinic staff, leaving patients with limited control over their medical records. Maternal information is primarily documented on physical cards that patients must carry for each visit. One respondent elaborated on this practice:

*“They have maternal cards that they bring to every visit. We also write the information in our registers on maternal and baby cards. However, they did not have access to the electronic system. ” (P2)*

A health information officer confirmed this restricted access.

*“Patients requesting their records would go to the ward where they were admitted to. With the patient's consent, clinicians would request access on their behalf. ” (P5)*

Moreover, this reliance on maternal cards impedes the effectiveness of referrals. Women must physically transport their records because fragmented facilities lack interoperable systems. Women must present their maternal cards and referral notes at higher-level facilities during the referral process. However, feedback from these facilities often fails to reach the referring clinics, disrupting continuity of care and leaving lower-level providers dependent on patients to relay critical information.

*“Currently, each clinic has its own data, and the systems are not connected. So, when patients transfer, they rely on their maternal cards, which contain all the information in the registers. Remember, we duplicate the information on the registers into the mother's maternal cards. ” (P4)*

“E*ach clinic has its own data, and the systems are disconnected from each other. Therefore, when patients transfer, they rely on their booklets. Patients take their cards with them when they are referred to another centre. We do not conduct deliveries here, but NE does, and if referred, as I said, they have to carry their maternal card.* ” *(P7)*

*“I must follow up and determine. Sometimes, I ask the patient if they can return after being at X, and regarding feedback, I have to follow up and find out what is transpiring. Sometimes, I have to ask the patient to return. Whatever the information they would have and what would have been done to manage the patient, I ask the patient to come. ” (P7)*

The emergent themes (T) associated codes (C), and their frequencies are presented in Table 4.

**Table 4.** Integrated themes, codes, and key findings.

Theme	Underlying codes(frequency)	Key findings
Data management issues	• Patient autonomy (C1, n=10)	Paper-based registers require manual duplication across registers; patients carry maternal/baby cards but have no digital access; electronic systems capture limited data (basic demographics, a maximum of three diagnoses); separate registers for family planning, antenatal care, postnatal care, and antiretroviral therapy; and there is a lack of data integration.
	• Duplicate effort (C2, n=24)	
	• Record inaccessibility (C3, n=5)	
	• Register redundancy (C4, n=20)	
	• Data quality and reporting (C5, n=3)	
Operational issues	• Disconnected clinics (C6, n=11)	Each clinic maintains independent records; patient transfers rely on physical maternal health cards; paper-based filing creates storage constraints; patients hold duplicate information that providers also record; and the electronic system is inaccessible to patients.
	• Paper referral reliance (C7, n=14)	
	• Inefficient record filing (C8, n=2)	
Data security and integrity	• Secure storage, data integrity and access control (C9, n=9)	Patient data are transmitted to the Ministry of Health repository without patient control; manual duplication risks transcription errors; clinic personnel can view all patient information (no individual access control); and patients lack verification or control mechanisms.

**Figure 3.**
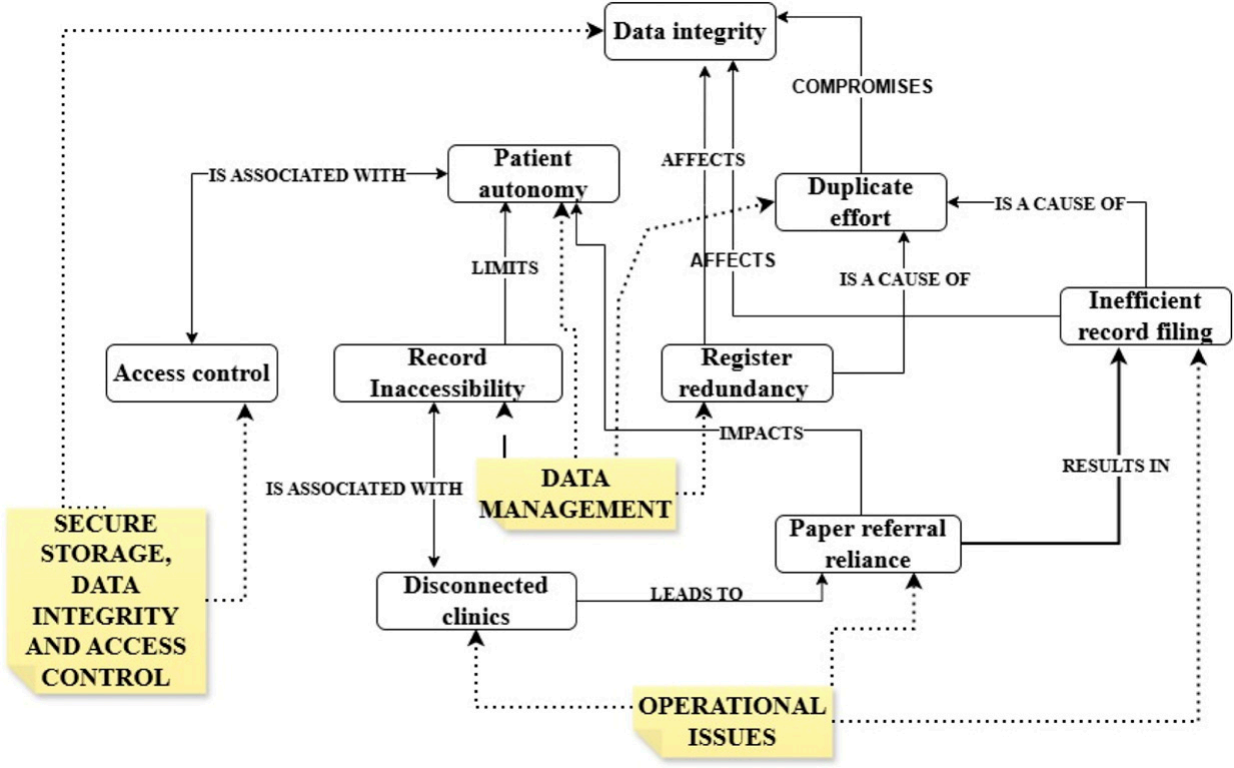
Challenges of care coordination in the current system.

Figure 3 illustrates the care coordination challenges within the current system, highlighting how fragmented data management practices, such as register redundancy, inaccessible records, and reliance on paper referrals, constrain patient autonomy and compromise data integrity. Inefficient record filing and duplicate efforts further exacerbate operational inefficiencies, while disconnected clinics limit the continuity of care.

### Interessement

In this study, interessement involved engaging healthcare professionals and fostering collaboration with external entities to align interests. The health information officer and nurses highlighted their collaborations with the Ministry of Health and the National AIDS Council (NAC). This collaboration is crucial to ensuring records are shared and the system meets the needs of various stakeholders.

*“I act as a bridge between the Ministry of Health, our partners such as the National AIDS Council and Open Fund for International Development, and the hospital itself. We are responsible for maintaining patient records and generating reports to track trends and patient outcomes. We produce monthly and weekly reports for the ministry and our partners… Well, it's a collaborative effort. The Ministry of Health oversees the healthcare system, whereas organisations such as the NAC support specific needs. Additionally, nurses, doctors, and lab technicians directly interact with patients and contribute to their medical records. ” (P5)*

The maternal card is a crucial boundary object that connects various network actors. The card is not just a passive record but an active participant in circulating and translating medical information. It moves between clinics, hospitals, and patients, bridging the gaps caused by disconnected healthcare facilities and ensuring continuity of care. Through this process, the card aligns diverse interests within the network, helping sustain coordinated care and confer agency. As evident from the interviews, the maternal card fills a critical gap created by disconnected clinics:

*“Currently, it is not connected. Each clinic has its own data. when patients transfer, they rely on their booklet.” (P1)*

*“The patients will only have access to their maternal and baby cards. ” (P7)*

*“The patients bring their antenatal, maternal cards so they have the same information that is in the registers written on their cards”…. “Patients take their card with them when they are referred to another centre.”(P8)*

### Enrolment

In this resource-constrained context, staff, patients, and technologies are “enrolled” into specific positions to keep the system functioning.^58^ Nurses perform dual roles as care providers and data clerks, balancing their clinical responsibilities with extensive documentation. Collectively, these roles ensure the network continues to function despite systemic constraints. One midwife explained:

*“I enter the data into the system and then transcribe the information from paper charts into the electronic register. All the nurses that deal with antenatal care are the ones that are responsible for entering this information*” *(P2).*

Patients are enrolled as carriers of their own data, maintaining continuity through maternal health cards. As a sister-in-charge described:

*“The patients bring their antenatal, maternal cards so they have the same information that is in the registers written on their cards... The person goes with their booklet. When they transfer, they use their books” (P1),* reinforced by a midwife:

*“We capture the information on their maternal health cards which they carry home... when patients transfer, they rely on their maternal cards which contain all the information in the registers*” *(P4).*

External partners, such as the National AIDS Council (NAC) and DREAMS, were enrolled as data consumers. One midwife noted:

*“We also have external partners that are interested in the information such NAC and the DREAMS programme” (P2),* and a nurse added, *“We also have other parties that sometimes access the records such as DREAMS. They are currently here am sure you saw their cars outside.* ” *(P3).*

### Mobilisation

The maternal booklet acts as a central but fragile spokesperson for the patient’s data, as noted by a nurse:

*“The challenge now becomes a shortage of resources as we don't have enough of those books, such that sometimes we need to have photocopies of those ANC booklets*” *(P6).*

The electronic system reports clinic data upward, but fails for lateral transfers. The sister in charge stated, *“Currently it’s not connected. Each clinic has its own data... So, someone relies on their book that they are given”(P1).* This was echoed by a midwife:

*“Currently, if a patient transfers from another clinic, we rely on their physical medical records*” *(P2*).

Within clinics, the paper register remains authoritative during disruptions: *“If the machine is down, we have to enter in the book... Every patient who comes is entered into the antenatal care register” (P7)*, and *“We do have the register, and that is the register which remains at the station” (P6)*.

### Informal workarounds

Across interviews and observations, the staff described a set of informal practices that sustained record-keeping despite fragmented systems. These include practices that duplicate entries across paper registers and electronic systems: “*The records are too many … I have to record the patient in all the books… 4 or 5*” *(P7).* Providers frequently revert to paper as the authoritative source during downtime: “*If the machine is not working, we have to enter it in the book”(P6).* Patients are also relied upon to transport maternal cards and referral notes between facilities. When referral feedback is missing, providers reported compensating through ad hoc follow-up: “*I have to follow up and find out what is transpiring …I have to ask the patient to come back”(P7).*

From an ANT perspective, the observed workarounds constitute mediators that stabilise fragile networks. When digital infrastructure fails to mobilise data, the burden of care continuity shifts to human actants and physical inscriptions. When electronic systems are unavailable, the paper register re-emerges as the authoritative inscription device that determines what constitutes legitimate clinical memory.

Furthermore, because women themselves are enrolled as couriers of clinical histories, the responsibility for maintaining continuity is effectively transferred from institutions to the individual patients. These dynamics reveal a fundamental tension between two competing logics: an *institutional logic* that prioritises reporting and upward data aggregation for the Ministry of Health and partner organisations, and a *clinical logic* that emphasises continuity and lateral information sharing during patient referrals.

## Identifying gaps in routine ANC care

**Figure 4.**
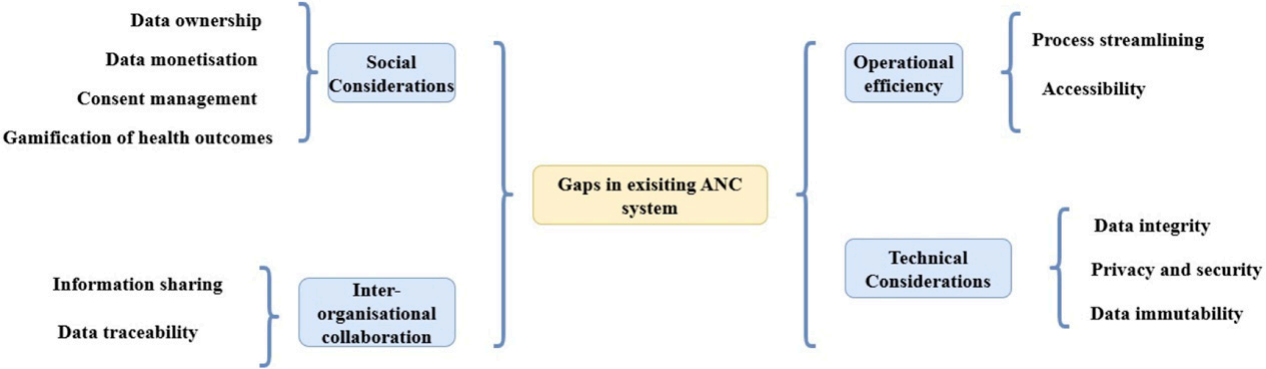
Gaps in the existing ANC system(Adapted from^40^).

As depicted in Figure 4, the current ANC system has several deficiencies that warrant a structured gap analysis to improve workflow and service quality. This study employed a modified version of the framework developed by Zein and Twinomurinzi,^40^ incorporating themes identified by^29^ in the literature. This integrated approach evaluates gaps across four key dimensions: technical improvements, social considerations, operational efficiency, and inter-organisational collaboration.

### Technical considerations

The current system relies heavily on paper-based maternal registers with manual data transcribed into electronic systems. This approach limits patient control over data access and is prone to errors, data integrity issues, tampering, and security breaches. Additionally, the lack of immutable audit trails increases the risk of data leakage and unauthorised modifications.

### Social considerations

The current maternal healthcare system disproportionately benefits hospitals and clinics that retain greater control over and access to patient data. In contrast, patients are generally limited to information recorded on paper-based maternal cards. Even facilities implementing electronic health record (EHR) systems often restrict direct patient access to medical information. This model limits patient autonomy and continuity of care, particularly during facility transitions, highlighting the lack of an equitable, patient-centred approach to data ownership.

### Operational efficiency

The current system is inefficient, requiring manual data duplication from paper registers into electronic records, wasting time and resources during consultations. This administrative burden distracts healthcare providers from focusing on direct patient care.

### Inter-organisational collaboration

The hierarchical nature of the healthcare system, which often relies on bottom-up referrals and paper-based medical records, creates significant barriers to collaboration among healthcare providers. This fragmentation limits timely access to patient information, undermining the delivery of evidence-based maternal healthcare.

## Conceptual architectural framework development

Based on the triangulation of empirical data from our thematic analysis and findings from our systematic literature review,^29^ this study proposes a novel 5-tier architectural framework to address the complexities of healthcare data management and utilisation. This triangulation was achieved by systematically mapping the requirements identified during stakeholder interviews, such as data portability and access control, alongside the gaps identified in the systematic literature review, such as the lack of patient data ownership, as explicitly detailed in Table 3.

This architectural framework is a conceptual design informed by stakeholder requirements identified in the field study; however, implementation and validation remain areas for future research. While the core workflow requirements, such as data portability and access control, are directly grounded in the empirical interview data, advanced architectural features, such as the NFT marketplace and tokenised incentive mechanisms, are conceptually derived from the literature,^32^ to address the systematic gaps in patient retention. Table 5 extends the mapping exercise already applied to literature-review insights in Table 3, this time translating codes derived from the empirical findings (Table 4) into architectural requirements and their corresponding framework elements. Three representative codes are shown; the remaining architectural elements were derived through the same procedure.

**Table 5.** Translation of interview-derived codes (Table 4) into architectural requirements and framework elements.

Interview-derived code (Table 4)	Implication/Requirement	Framework element in conceptual design
Duplicate effort (C2); Record inaccessibility (C3)	A single, authoritative, portable record that removes manual re-entry	Data Layer (on-chain hash + off-chain store)
Disconnected clinics (C6); Paper-referral reliance (C7)	Automated inter-facility data portability during referral	Referral service, Business/Application Logic Layer
Patient autonomy (C1); Record inaccessibility (C3)	Patient-controlled, revocable consent to grant/withdraw access	Patient smart contract, Contract Layer

**Figure 5.**
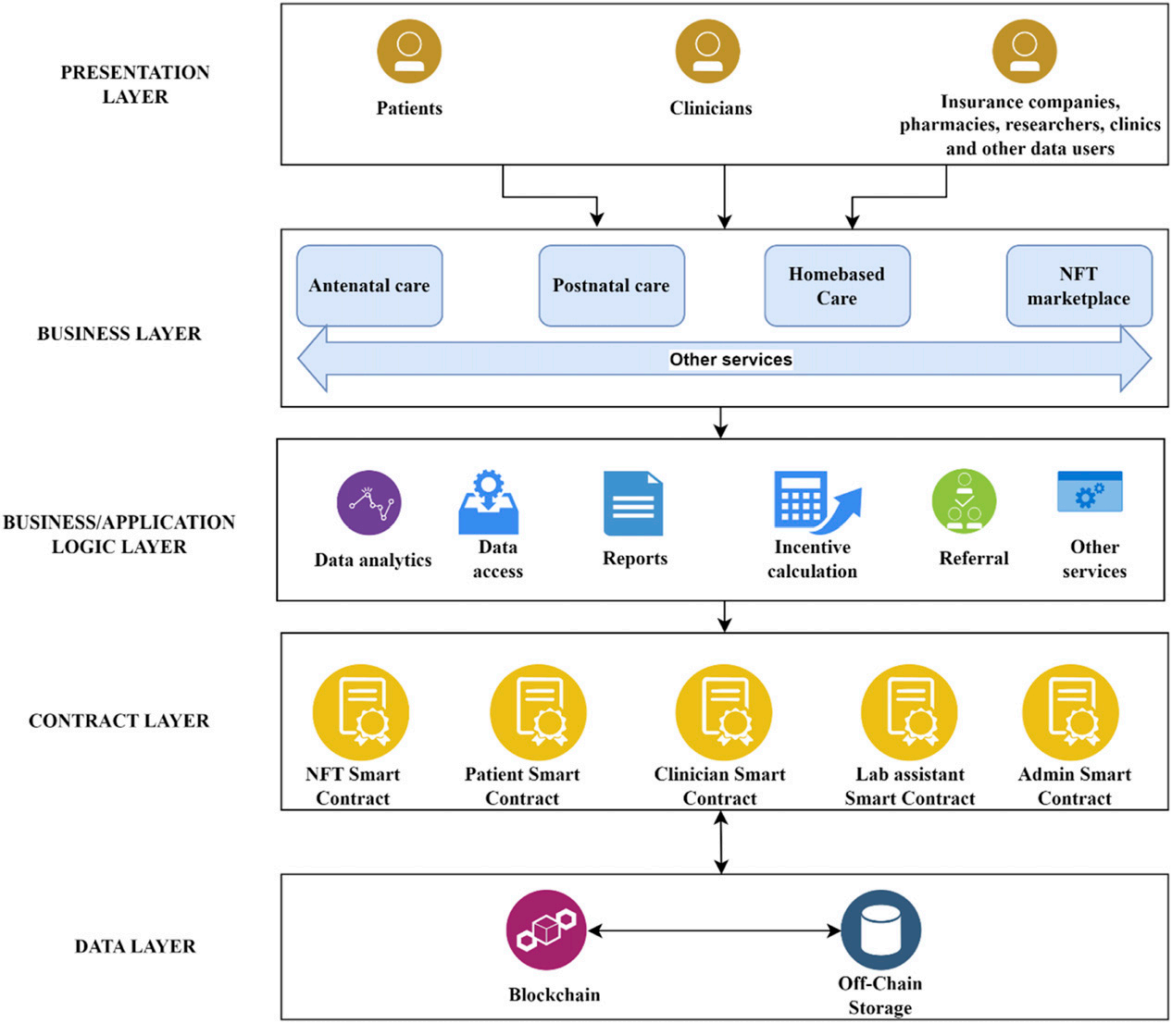
Five-tier architectural framework.

The conceptual architecture comprises distinct layers, each fulfilling a specific function. The Presentation Layer is the user interface that provides access to various healthcare services. The Business Layer encapsulates the core business logic, including patient care, clinical services, and NFT marketplace functionalities. The Business/Application Logic Layer houses data analytics, access, reporting, incentive calculation, referral services, and other essential operations. The Contract Layer incorporates smart contracts for NFT management, patient data, clinician interactions, laboratory assistant tasks, and administrative functions. Finally, the Data Layer leverages blockchain technology for secure and transparent data storage, complemented by off-chain storage to efficiently handle large datasets. Ultimately, the layered architecture facilitates data privacy and interoperability while introducing mechanisms that encourage responsible data sharing among healthcare stakeholders. The proposed architectural framework is illustrated in Figure 5, and the functionality of each layer is described as follows.

### Data layer

Standardised data formats and communication protocols are essential to ensure data accessibility, interoperability, and system compatibility (C3). Clearly defined interfaces and structured data exchange mechanisms enable seamless documentation and efficient information sharing across healthcare systems. Robust and secure storage solutions are also critical for maintaining data integrity.^59^ Blockchain-based solutions, particularly NFTs, potentially offer tamper-resistant approaches to data management (C9). By leveraging the immutability and transparency of blockchain technology, NFTs can securely reference and control access to sensitive medical information, protecting it from unauthorised access or alteration. Off-chain storage is utilised for large datasets to enhance privacy and efficiency (C3). This reduces the load on the blockchain because on-chain data storage is generally expensive and inefficient. Instead, the blockchain stores only small, immutable data elements, such as metadata or cryptographic hash values, which serve as verifiable pointers to the encrypted data stored off-chain.^29,60^

### Contract layer

The Contract Layer stores and manages smart contract logic, enabling the automated execution of predefined actions on the blockchain network. A smart contract is a self-executing script that runs on a blockchain when specific conditions are met.^28^ Smart contracts can support data access control, authentication, and transaction auditing in healthcare. NFTs have been proposed to reduce the risks associated with data breaches while enhancing patient autonomy over personal health information (C1, C9). NFTs can serve as verifiable and secure access tokens that reference patient records rather than the data itself. This would allow patients to retain greater control over how their medical data is shared, enabling selective disclosure to trusted healthcare providers and facilitating more informed decision-making (C1). This framework links patient data to NFTs through an NFT smart contract. Role-based access is enforced using additional smart contracts tailored to different system actors. For instance, patients can use a dedicated smart contract interface to grant or revoke access to their data in response to clinicians’ requests. Health administrators can manage system users, such as clinics, hospitals, patients, and healthcare staff, by invoking administrative smart contracts to assign roles and permissions to them. Lab technicians and health information officers can contribute to or retrieve authorised health information as permitted by patient or system policies. Ultimately, this model is designed to position patients as data custodians, with NFTs serving as decentralised, auditable access keys intended to provide permissions and provenance (C1).

### Business/application logic layer

The application logic layer implements the business rules and processes that govern the application’s behaviour, defining core functionalities such as calculations, data transformations, and reporting (C5, C8). This layer supports the underlying operational mechanisms of the NFT marketplace and incentive calculations while ensuring data integrity and access control (C9) to incentivise patients to share data with authorised third parties and attend antenatal and postnatal care appointments. It interacts directly with the data layer to facilitate efficient data retrieval and seamless transfer of patient records between clinics during referrals, addressing the challenge of disconnected facilities(C6).

### Business layer

The business layer encapsulates the core functionalities of patient services, including antenatal, postnatal, and home-based care. Additionally, drawing on concepts from the broader literature, this layer proposes a conceptual model of an NFT marketplace. Because medical data holds significant value for research institutions and insurance companies, this module is hypothesised to incentivise patients to safely share data and adhere to scheduled appointments by allowing them to trade tokenised data assets securely.^32^

### Presentation layer

The presentation layer serves as the user interface and provides access to various healthcare services. Different user groups, such as doctors, nurses, and third-party researchers, interact with the system based on their specific needs. By visualising the rules enforced by the contract layer, this interface is designed to potentially enable patients to actively maintain control over their information by granting or denying access to specific individuals or organisations governed by smart contracts.

### Illustrative workflow: A referral scenario

In the proposed design, a maternal record is instantiated at the first antenatal visit as an NFT: the encrypted clinical payload resides off-chain, while only its hash and metadata are committed on-chain (C3, C9). Subsequent visits add versioned off-chain entries, retaining prior versions rather than overwriting them (C2, C5). At referral, access is delegated to the receiving clinician for a bounded period through the patient smart contract, removing reliance on a physically transported maternal card (C1, C6, C7); the receiving facility then obtains a complete, verifiable record via the referral service without depending on the patient to relay information (C6). Access subsequently lapses or is revoked, and each grant, retrieval and update is retained in an immutable audit trail. These interactions map directly onto the deficiencies identified in the current system: record duplication, disconnected facilities, reliance on paper referrals, and limited patient control, and specify how the architecture would reconfigure the maternal-care network characterised through the ANT analysis. The scenario is presented to specify intended design behaviour; empirical validation of these interactions through usability and implementation studies remains future work (see Limitations and Future Research).

### Implementation and governance considerations

The practical deployment of this framework requires addressing several technical and organisational challenges.

### Blockchain architecture

The system is envisioned as a permissioned consortium blockchain, where verified healthcare institutions (hospitals, clinics, and the Ministry of Health) operate network nodes. This approach was designed to balance the need for patient privacy with institutional accountability. Unlike public blockchains, a permissioned model has the potential to reduce transaction costs and energy consumption while maintaining auditability. Platforms such as Hyperledger Fabric or Polygon PoS represent candidate technologies, although the final selection would depend on pilot-phase performance testing and stakeholder consultations. Governance of the consortium would require transparent decision-making mechanisms; approaches such as quadratic voting have been proposed to balance stakeholder influence and prevent institutional capture in blockchain-based healthcare systems.^61^ Responsibility for the smart-contract lifecycle upgrades, patching of identified vulnerabilities, and deprecation would also need to be formally allocated within the consortium’s governance charter, since deployed contracts are difficult to alter, and unmanaged contract logic could otherwise become a single point of organisational failure.

### Identity and key management

In settings where digital literacy and smartphone access are limited, managing patient identities is a critical challenge. A custodial wallet model is proposed as an interim solution in which healthcare facilities securely store encrypted private keys on behalf of patients who lack the technical capacity to self-custody their keys. Patients would then be authenticated using existing national identification systems supplemented by biometric and point-of-care verification. Lost key recovery would be managed through a multi-signature protocol, requiring approval from two of the three authorised designated parties, for example, the patient’s nominated guardian and two healthcare providers, to restore access without creating single-point vulnerabilities.

### Consent withdrawal and data correction

While blockchain data are immutable by design, the proposed architectural framework employs on-chain and off-chain storage to enable patients to withdraw consent or correct errors. The blockchain stores NFT metadata and access permissions, whereas the actual medical records reside in encrypted off-chain storage, such as institutional databases or distributed systems like IPFS. Consent withdrawal revokes on-chain access tokens, rendering previously authorised off-chain data inaccessible, whereas data corrections are implemented by appending new record versions with appropriate versioning metadata rather than altering historical entries. This preserves auditability while accommodating the clinical updates.

### Regulatory compliance

From a data privacy perspective, this decentralised approach potentially serves as a mechanism for regulatory compliance. By embedding immutable audit trails and consent provenance directly into the NFT architecture, the framework natively supports the principles of stringent data protection laws, such as the EU General Data Protection Regulation (GDPR). It potentially enables patients to automatically track where and with whom their data has been shared. Crucially, this may allow healthcare providers to satisfy these advanced privacy requirements without changing their systems to be compliant. Deployment in Zimbabwe would fall under the Cyber and Data Protection Act [Chapter 12:07] (2021), administered by POTRAZ as the Data Protection Authority,^62^ together with SI 155 of 2024, which requires data controllers to register and appoint a data protection officer.^63^ The Act mandates lawful, purpose-limited processing, heightened safeguards for health and biometric data, breach notification, and restrictions on cross-border data transfers. The proposed architecture is broadly congruent with these duties, immutable audit trails and on-chain consent provenance support transparency and accountability, while off-chain encryption addresses the security obligations, but two tensions require attention: the Act’s data-correction rights must be reconciled with blockchain immutability (addressed here by confining immutable content to metadata and access tokens), and node siting must be assessed against the transfer restrictions. This reinforces the need for regulatory feasibility assessment before any pilot.

### Security considerations

Given the sensitivity of maternal health information, potential vulnerabilities must be considered. The transition to a decentralised network introduces novel technical and sociotechnical vulnerabilities. Table 6 outlines the primary vulnerabilities anticipated with the proposed architectural framework and corresponding mitigation strategies.

**Table 6.** Proposed mitigation strategies for possible threats.

Security domain	Vulnerability/threat	Proposed mitigation strategy
Network consensus	Consensus-level attacks (e.g. node collusion in permissioned networks).	Implementation of the Practical Byzantine Fault Tolerance (PBFT) mechanism and multi-stakeholder governance oversight.^64^
Smart contracts	Technical exploits, such as re-entrancy or access control flaws.	Use of formal code verification, third-party security audits and adherence to established standards such as OpenZeppelin.^65^
Off-chain storage	Unauthorised access or data leakage in external repositories (IPFS/databases).	Mandatory end-to-end encryption(AES-256) with patient-controlled decryption keys and strict regulatory compliance.^66^
Identity and access	Key management risks (lost credentials) and insider threats from facility staff.	Use of Hardware Security Modules(HSM), link-based biometric recovery and immutable audit logging for all custodial actions.^67^

## Discussion

In this study, we developed and motivated an NFT-based architectural framework for electronic health record management in maternal healthcare in resource-constrained settings. The discussion is organised around the study’s three research objectives: (1) identifying deficiencies in current antenatal and postnatal record management, (2) examining how key actants interact within these practices, and (3) conceptualising how an NFT-based architectural framework can be designed to address these challenges. Actor-Network Theory (ANT) is applied to interpret the findings, with its four moments of translation, problematisation, interessement, enrolment, and mobilisation providing a lens for analysing how NFTs can realign stakeholder interests, strengthen patient agency, and improve system efficiency.

### Problematisation

The findings from the literature review and interviews converge on several persistent challenges, including fragmented paper-based systems, duplication of information, limited patient control, poor data quality, and weak referral mechanisms.^5,14,55^ These issues constrain patient agency, undermine data integrity and diminish operational efficiency. Within the ANT perspective, such challenges are framed as points of misalignment, where patients, healthcare workers, health information systems, and institutional policies fail to stabilise around shared objectives. Although some records are captured electronically, access remains limited to clinic staff, leaving patients without meaningful control over their health data. Patient agency, or patient-centred healthcare, in which patients are central to decisions about their care, can only be realised if individuals have adequate access to their information.^22,23^ To address this gap, the proposed framework conceptualises access to maternal healthcare data as NFTs, empowering patients with enhanced control over their records.^52,53^ The proposed NFT-based architectural framework directly addresses these deficiencies by conceptualising maternal health data as patient-owned digital assets; by shifting ownership from institutions to patients, records become portable, verifiable, and resistant to corruption or unauthorised alteration. This reframes the patient not as a passive data subject but as a central actor with agency in the healthcare system. In operational terms, NFTs confer data ownership to patients, enabling them to monitor and regulate access while ensuring their privacy. Integrating an NFT marketplace into the business layer facilitates controlled data sharing without intermediaries, allowing patients to monetise and trade their health information by minting it as NFTs.^32^ Developing legal and ethical frameworks will be necessary to realise this potential safely and effectively.

### Interessement

The second research question examined how different actants (patients, clinicians, institutions, and technologies) interact within current systems. Stakeholders articulate diverse, and at times divergent, priorities. Clinicians emphasise efficiency, accuracy, and timely access to reliable information, whereas patients prioritise secure access to and ownership of their records. Observations in select facilities further revealed that institutions are primarily concerned with their reporting obligations and compliance requirements. In this context, smart contracts serve as intermediaries that mediate these interests. Patients can grant or revoke access to their records, thereby preserving their agency and privacy, while healthcare providers benefit from timely and authorised access to accurate data. Moreover, token-based incentive mechanisms, such as rewards for attending consultations, are hypothesised to align patient behaviour with broader public health objectives, thereby potentially addressing the persistent challenge of low retention rates.^56^ From an ANT perspective, interest is achieved by demonstrating to each actor that their priorities are accommodated within the NFT-enabled system. However, accommodating divergent interests introduces tensions that must be explicitly addressed. While token-based incentives may align patient behaviour with public health goals, they risk creating coercive pressure on economically vulnerable women. Similarly, empowering patients to monetise health data through an NFT marketplace could enable exploitation if governance mechanisms do not protect against asymmetrical power dynamics. These concerns are examined further in the following section, after the framework’s operational design is described.

### Enrolment and mobilisation

Registers facilitate information flow but constrain efficiency, as clinicians remain custodians of records while patients assume a largely passive role. The proposed NFT framework aims to redefine these roles by enrolling patients as active data owners, positioning healthcare workers as authorised custodians with time-bound access, and establishing NFTs as immutable, transferable tokens of medical history. Institutional actors, such as health information officers, are repositioned to oversee system integrity and compliance, rather than solely managing records. High-quality antenatal and postnatal care (ANC and PNC, respectively) requires timely access to accurate and reliable data. In this regard, NFT-based health information systems provide a promising alternative to traditional methods by prioritising data security, privacy, and immutability.^28^ Through enrolment, the healthcare network is thus restructured so that both human and nonhuman actors contribute to a more balanced, secure, and efficient care ecosystem.

Mobilisation within the current ecosystem remains weak, characterised by poor inter-facility communication and fragmented reporting. The NFT framework was designed to address this limitation by introducing a common infrastructure, a distributed ledger that connects patients, providers, and facilities within a unified system. This shared platform theoretically enhances cohesion by reducing duplication and ensuring that all stakeholders interact with a single, verifiable version of patient records. Crucially, mobilisation is not only technological but also behavioural. Its success depends on whether patients and providers trust the system and perceive its adoption as having tangible value. Incentive structures embedded within the NFT marketplace, such as controlled data sharing and the potential monetisation of anonymised health data, offer sustainable motivation for engagement. The framework has the potential to reinforce mobilisation by binding diverse actors into an interconnected, self-sustaining healthcare network. Before elaborating on the implementation challenges, it is important to situate the proposed NFT-based framework within the landscape of existing digital health solutions for maternal care in resource-constrained settings. Table 7 compares the proposed approach with established alternatives across the key dimensions.

**Table 7.** Comparison of alternative approaches for maternal health record management in resource-constrained settings.

Approach	Key features	Advantages	Limitations	Patient agency
Centralised EHRs (e.g., OpenMRS)	Institutional databases; clinician-controlled access	Proven, cost-effective, widely deployed in Africa; mature technology	Institutional control, limited portability, and interoperability challenges across systems	Low - patients are data subjects, not owners
Federated EHR + FHIR	Distributed institutional systems with standardised data exchange	Enables cross-facility data sharing; preserves institutional autonomy	Centralised servers create single points of failure; patients are dependent on institutional cooperation	Low-Medium - access contingent on institutional participation
Permissioned Blockchain (e.g., Hyperledger Fabric)	Distributed ledger with role-based access control (RBAC)	Immutability; auditability; no single point of failure	Binary permissions (granted/denied); lacks individual asset ownership concept	Medium - access control but not ownership
Self-Sovereign Identity (SSI)	Decentralised patient identity and credential management	Patient-controlled credentials; privacy-preserving	No native support for tokenised incentives or granular consent tied to specific encounters	Medium-High - identity control but limited data governance
NFT-Based Framework (Proposed)	Tokenised health records as patient-owned digital assets	Individual asset ownership; tokenised consent with provenance; integrated incentive marketplace	High technical complexity; requires stakeholder education; governance challenges in resource-constrained settings	High - patients as asset owners with control over sharing and monetisation

As shown in Table 7, the NFT-based approach offers three distinct capabilities: (1) individual asset ownership, reframing patients as asset holders rather than data subjects; (2) tokenised consent with immutable provenance, allowing granular, auditable permissions beyond binary access control; and (3) integration of incentive marketplaces that reward adherence to care protocols without requiring cash transfer infrastructure. However, these advantages come with trade-offs. NFT frameworks introduce greater technical complexity, require more stakeholder education, and demand robust governance structures that may be difficult to establish in resource-constrained settings. The choice between these alternatives should be informed by pilot testing, cost-benefit analysis, and meaningful co-design with patients and providers.

### Ethical implications and equity safeguards

The capabilities enabled by NFT-based data ownership, particularly tokenised incentives and marketplace-based data sharing, raise ethical concerns that warrant explicit consideration before deployment. Safeguards should prioritise non-monetary rewards (such as priority appointment scheduling and achievement badges), design incentive structures through community consultation to align with local values and maintain voluntary participation by offering opt-out options that do not compromise care quality.

The NFT marketplace concept, while designed to empower patients to control data sharing, introduces potential for exploitation where third parties (pharmaceutical companies, insurance providers, research institutions) pressure economically vulnerable women to commodify their health information. Governance mechanisms must prohibit the sale of commercial data without informed consent, require ethics board approval for all research studies, and ensure benefit-sharing principles that ensure participants receive fair compensation and community-level benefits from aggregated data applications.

The framework also risks exacerbating existing inequalities if access depends on smartphone ownership, internet connectivity, or digital literacy. Women in rural areas, those with limited education, or those lacking formal identification may be systematically excluded. Mitigation strategies include facility-based access via tablets or kiosks, USSD-based fallback systems for feature phones, user interface localisation in local languages, and ensuring that fundamental care quality does not depend on platform participation. Full digital inclusion will require parallel investments in infrastructure and education extending well beyond the healthcare system itself. These ethical considerations underscore that technology alone cannot solve systemic inequities and may inadvertently deepen them if deployment is not accompanied by deliberate, equity-focused design and governance.

## Limitations and future research

This study has some limitations that must be acknowledged. The proposed architecture remains a design concept informed by stakeholder interviews and literature review, not an empirically validated system. The benefits described, improved interoperability, enhanced patient agency, and strengthened referral mechanisms are hypothesised outcomes based on the technical properties of blockchain and NFTs, not demonstrated effects.

A scope limitation is the absence of direct patient interviews. The study focused exclusively on healthcare providers and health information officers to establish technical requirements. Critically, because patient perspectives were not directly captured, the framework’s central claim, that tokenised ownership will enhance patient agency, currently rests on provider-reported and literature-derived inference rather than on evidence from pregnant women themselves. The anticipated agency, trust and usability benefits should therefore be read as design hypotheses for the co-design phase, not validated outcomes; patients may prioritise different features or encounter barriers that the present provider-focused account cannot anticipate. For a framework centred on patient agency, future research with the resultant NFT artefact should include comprehensive assessments of patient adoption and acceptance. Pregnant women’s perspectives on digital health tools, consent interfaces, and trust in tokenised systems remain unexplored. Future research must prioritise participatory co-design involving women as active design partners to ensure the framework aligns with their needs, values, and lived realities.

The findings reflect challenges common across resource-constrained settings: fragmentation, limited patient control, and weak referral mechanisms. Addressing these context challenges requires a concrete, phased research agenda. First, patient usability studies must be conducted through participatory workshops with pregnant women and mothers to evaluate consent interfaces, digital trust, and technological accessibility. Second, regulatory feasibility assessments are necessary to navigate national data protection laws, institutional governance frameworks, and ethical compliance before deployment. Third, comparative pilots with non-NFT systems (such as traditional centralised EHR platforms) should be implemented in clinical settings to empirically evaluate the framework’s actual impact on record completeness, referral completion rates, visit adherence, and patient-reported agency. Finally, to address technical and infrastructure uncertainties, longitudinal implementation studies must investigate the framework’s scalability, cost sustainability, and the effectiveness of equity-focused deployment strategies, such as facility-based kiosks or USSD-based fallbacks, under real-world conditions to ensure reliable access for all women.

## Conclusion

This study aimed to identify actant interactions and systemic deficiencies in the current management of medical records for antenatal and postnatal care, thereby informing the design of an NFT-based framework to address these challenges. A qualitative case study was conducted across primary healthcare sites in a metropolitan area and one referral centre in Zimbabwe. The research adopted a qualitative approach to explore the intersection of blockchain technology, NFTs, and maternal healthcare. ANT, particularly the concept of translation moments, was employed to guide the thematic analysis and systematically identify stakeholder roles, system limitations, and potential areas for technological intervention.

The findings underscore the urgent need to reduce reliance on paper-based records, which are prone to storage limitations, inefficiencies, and data loss. In response to these challenges, this study proposed a conceptual NFT-based framework to enhance workflow efficiency, improve access to patient information, and promote interoperability among healthcare providers, thereby supporting improved referral processes and continuity of care. However, the benefits described remain hypothetical; empirical validation through co-design, pilot implementation, and rigorous evaluation is essential before adoption can be recommended.

An emerging implication of this model is its potential to create new data value chains, in which patients could grant entities such as researchers, insurance companies, or pharmaceutical firms’ permission in exchange for defined benefits. Such arrangements would require robust ethical, legal, and consent frameworks to safeguard patient rights and prevent exploitation, concerns that must be addressed through participatory governance design and regulatory consultation before any deployment. Realising and validating the framework will require a staged programme: prototype development, usability evaluation with pregnant women and providers, comparative pilot deployment against existing EHR systems in clinical settings, and economic evaluation of sustainability under resource constraints, followed by phased scale-up. The following section discusses the theoretical and practical contributions in more detail.

### Theoretical contributions

Theoretically, this study advances understanding of blockchain and NFT applications in maternal healthcare through three contributions. First, it proposed a novel conceptual framework that integrates NFT-based data ownership with patient-centred care principles in resource-constrained settings, extending existing blockchain-EHR models to address the specific challenges of fragmented maternal health records. Second, by employing ANT, the study demonstrates how digital health interventions can be analysed as socio-technical networks in which human actors (patients, providers, administrators) and non-human actors (smart contracts, NFTs, blockchain infrastructure) must be aligned to achieve sustainable mobilisation. This approach moves beyond purely technical descriptions to examine power dynamics, informal practices and competing institutional logics that shape health information systems. Third, the framework contributes to policy discourse on strengthening healthcare systems to ensure universal health coverage and access to quality services, aligning with Sustainable Development Goal 3 (SDG 3), while acknowledging the governance, equity, and sustainability challenges inherent in blockchain-based interventions.

### Practical contributions

From a practical perspective, this study offers a design blueprint for improving maternal healthcare data management in resource-constrained settings through blockchain technology and NFT-based access tokenisation. Upon pilot implementation, the framework could yield several practical benefits. A key potential implication is the promotion of patient agency, whereby individuals gain greater control over who accesses their medical information, shifting from passive data subjects to active participants in their healthcare journey.

For healthcare practitioners and policy makers, this study provides a structured roadmap for exploring NFT adoption in maternal health, including specific technical architecture (five-tier framework), governance considerations (permissioned consortium blockchain, custodial wallet management), and security requirements (threat modelling, encryption protocols). The framework’s design addresses real-world constraints identified through stakeholder engagement, limited digital literacy, unreliable connectivity, and resource scarcity, thereby grounding technological proposals in operational realities.

The study further proposed an incentive-based mechanism to encourage patient engagement, such as issuing redeemable tokens for attending consultations or completing recommended care protocols. If implemented ethically, such initiatives motivate adherence to WHO-recommended antenatal and postnatal schedules, promote healthier behaviours, and support more comprehensive health data collection. However, as discussed in the ethical considerations and limitations section, the feasibility, acceptability, and equity implications of these mechanisms require empirical investigation through participatory co-design with pregnant women and mothers before deployment.

## Data Availability

The data that support the findings of this study are available from the corresponding authors upon reasonable request and in accordance with research ethics committee policies and privacy regulations.*
